# Looking beyond the veil of the European crisis - the need to uncover the structural causes of health inequalities

**DOI:** 10.1186/s12939-016-0329-5

**Published:** 2016-03-01

**Authors:** Antonio Escolar Pujolar, Amaia Bacigalupe, Miguel San Sebastian

**Affiliations:** Delegación Territorial de Igualdad, Salud y Políticas Sociales, Junta de Andalucía, Cádiz, Spain; Department of Sociology, University of the Basque Country (UPV/EHU), Leioa, Spain; Epidemiology and Global Health, Department of Clinical Medicine and Public Health, Umeå university, Umeå, Sweden

We are happy to announce the second cluster of papers around the economic crisis and its impact on health in Europe in our journal, the International Journal for Equity in Health. Nine empirical articles and two commentaries are included in this volume. This important amount of research points to the relevance of continuing to monitor the health impacts of the economic crisis in the European region.

While certain macroeconomic recovery has been observed in the last couple of years, the economic situation remains unstable, particularly for the Mediterranean countries which still face numerous economic challenges. High unemployment rates, temporary contracts, low salaries and diminished social benefits, among others, are current social determinants which will continue deteriorating the health of the European population [1].

After our first editorial two years ago [2], we have witnessed how the European region has moved from the crisis situation and settled into a period of stagnation. Aiming to overcome the crisis under a neoliberal development model, most of the governments across the region implemented austerity policies based on regressive tax structures and deep spending cuts, particularly to public services such as education, health and social security. These policies may prove to be shortsighted and only contribute to the erosion of mechanisms that reduce inequality and enable equitable growth [3]. As the economist Paul Krugman recently wrote in the Guardian “Since the global turn to austerity in 2010, every country that introduced significant austerity has seen its economy suffer, with the depth of the suffering closely related to the harshness of the austerity” [4]. Now, leading proponents of austerity, such as the International Monetary Fund (IMF), are beginning to recognize that these harsh measures have not led to the expected results, and have harmed both growth and equality [5]. Will this mean a change in European policies in the near future?

To complicate the scenario, the next coming years are going to be marked by new and old realities, making the political context of the economic crisis potentially more complex and prolonged: i) European nations are still suffering record levels of long-term and youth unemployment, with a generation of young people facing years of joblessness to come, and many countries, particularly those in Southern Europe experiencing tremendous brain drain [6, 7]; ii) More than one million migrants fleeing from war zones (mainly Syria, Iraq and Afghanistan) have entered the European Union (EU). This will have economic implications for the European economy [8], in addition to the potential risks of growing xenophobia and violence against migrants; iii) The acts of terrorism in France in 2015 might lead to a loss of individual liberties on behalf of higher security, creating new elements of social stress; iv) The current Transatlantic Trade and Investment Partnership (TTIP) trade negotiations between the EU and United States has already raised several concerns regarding serious threat to national governments’ right to regulate, and protect public health services, consumer rights, the rights of workers or the to keep policies safeguarding the environment [9, 10]; v) Finally, the World Health Organization considers climate change as the greatest threat to global health in the 21st century [11]. The Paris agreement signed on 12 December 2015 commits signatories´ countries to limit global warming to well below 2 degrees Celsius [12]. Though positive, a word of caution regarding its achievement is necessary given the current economic development model that promotes CO^2^ emissions. How will these events impact the health of the different populations that live in Europe and what does this mean for the growing levels of inequalities among the different social groups?

Our invited commentary from Greece illustrates that government efforts have not been enough to protect the most vulnerable, and so health inequalities persist [13]. The eight articles included here compared health and health inequalities in pre and during crisis periods using different databases and health outcomes, six of them coming from Spain.

There are important issues related to which health outcome(s) could be more sensitive to capturing the effects of the economic crisis. Data shows that self-rated health has not worsened during this period, and in some cases it may have even improved during the crisis period, as observed in the studies by Arroyo et al. in Spain and Adebe et al. using European data [14, 15]. The situation is, however, completely different when other health outcomes more closely related to chronic diseases are considered. This is supported by four Spanish studies included in this cluster which have found a deterioration of population´s health after the beginning of the crisis in diabetes, cardiovascular diseases [16] and in different dimensions of mental health [17–19]. Additionally, an increase in the suicide rate from 2010 in Catalonia, Spain was found in working-aged women (16–64 years) living in municipalities with 10,000 or more inhabitants [20].

The different impact of the crisis on social groups is the second important finding we would like to highlight. Low educated groups, children from low socioeconomic families [17] and groups with different employment status [18], those with housing difficulties [19] or women in working age [20] are some of the vulnerable groups identified here, and it is their health that has been more severely affected. These findings underscore the importance of collecting disaggregated data to be able to identify the distinct health experiences of the crisis.

As a final reflection, the vision we get from the papers received is partial, and to some extent minimalist but they highlight the need to add a more holistic view. The importance of visualizing the political and economic framework in which these small realities emerge, and the need to better understand the historical dynamics that lead to this multiplicity of effects should serve as a guide for future research. Our suggestion is to not only continue monitoring the current economic crisis and its impact on health inequalities but to clearly uncover the consequences of the ruling neoliberal development model. Research on social inequalities in health should be considered in more structural terms, showing in a clearer light the existing link between everyday life and the policies emanating from the dominant socio-economic model that promotes such start austerity measures. This may lead to gaining a less cyclical and a more radical perspective in identifying the causes of the causes. We look forward to exploring this for our next cluster of papers.
